# Health in refugees and migrants who self-identify as sexual or gender minority

**DOI:** 10.1093/eurpub/ckac131.520

**Published:** 2022-10-25

**Authors:** E Mattelin, F Fröberg, L Korhonen, AR Khanolkar

**Affiliations:** 1 Barnafrid, Linköpings University, Linköping, Sweden; 2 School of Life Course and Population Sciences, King’s College London, London, UK; 3 Department of Global Public Health, Karolinska Insitute, Stockholm, Sweden; 4 Center for Social and Affective Neuroscience, Linköping University, Linköping, Sweden; 5 Department of Child and Adolescent Psychiatry, Linköping University, Linköping, Sweden

## Abstract

**Background:**

In 2019, 80 million individuals were forcibly displaced. Yet, there is little knowledge on the health of refugees and migrants who self-identify as sexual- or gender minority (SGM). The aim was to examine health and health-related behaviors in refugee and migrant individuals who identify as SGM, and in comparison, to their heterosexual peers.

**Methods:**

This study included 168,952 persons aged 16-84 who answered the Swedish National Public Health Survey in 2018 and 2020. Participants were grouped into White heterosexual, White SGM, migrant heterosexual, migrant SGM, refugee heterosexual, and refugee SGM. Outcomes included mental health (f.e. mental ill-health, suicidal ideation), general health, risky behaviors (f.e drug and alcohol use), and experiences of violence. Associations were analyzed using logistic and linear regression adjusting for sex, age, and educational level.

**Results:**

Being an SGM, regardless of refugee or migrant minority status, was associated with worse general health and mental ill-health compared to heterosexual peers including suicidal ideation in refugee SGM (OR 2.42, 95 % CI 1.44-4.08). Both refugees and migrants had for example lower odds of drug and risk alcohol use compared to heterosexual peers but higher odds of risk gambling (OR 1.88, 1.49-2.37 for refugee SGM). Transgender refugees had high odds for risk gambling (OR 8.62, 1.94-38.40) and exposure to physical violence (OR 7.46, 2.97-18.70).

**Conclusions:**

In this national population-based study, SGM have worse mental and general health regardless of being refugee or migrant minority. SGM refugees did not have worse health compared to migrant and White SGM and their heterosexual peers. While more research is needed, our study shows the need for public health personnel to be aware of potentially worse health and adverse experiences in SGM individuals, regardless of ethnic origin including refugees.

**Key messages:**

• Our study provides evidence for poorer health outcomes in diverse SGM-groups.

• Policies tackling health in refugees and SGM are still inadequate.

